# Cytoplasmic Fungal Lipases Release Fungicides from Ultra-Deformable Vesicular Drug Carriers

**DOI:** 10.1371/journal.pone.0038181

**Published:** 2012-05-29

**Authors:** Gero Steinberg

**Affiliations:** Biosciences, College of Life and Environmental Sciences, University of Exeter, Exeter, United Kingdom; University of Wisconsin – Madison, United States of America

## Abstract

The Transfersome® is a lipid vesicle that contains membrane softeners, such as Tween 80, to make it ultra-deformable. This feature makes the Transfersome® an efficient carrier for delivery of therapeutic drugs across the skin barrier. It was reported that TDT 067 (a topical formulation of 15 mg/ml terbinafine in Transfersome® vesicles) has a much more potent antifungal activity *in vitro* compared with conventional terbinafine, which is a water-insoluble fungicide. Here we use ultra-structural studies and live imaging in a model fungus to describe the underlying mode of action. We show that terbinafine causes local collapse of the fungal endoplasmic reticulum, which was more efficient when terbinafine was delivered in Transfersome® vesicles (TFVs). When applied in liquid culture, fluorescently labeled TFVs rapidly entered the fungal cells (T_1/2_∼2 min). Entry was F-actin- and ATP-independent, indicating that it is a passive process. Ultra-structural studies showed that passage through the cell wall involves significant deformation of the vesicles, and depends on a high concentration of the surfactant Tween 80 in their membrane. Surprisingly, the TFVs collapsed into lipid droplets after entry into the cell and the terbinafine was released from their interior. With time, the lipid bodies were metabolized in an ATP-dependent fashion, suggesting that cytosolic lipases attack and degrade intruding TFVs. Indeed, the specific monoacylglycerol lipase inhibitor URB602 prevented Transfersome® degradation and neutralized the cytotoxic effect of Transfersome®-delivered terbinafine. These data suggest that (a) Transfersomes deliver the lipophilic fungicide Terbinafine to the fungal cell wall, (b) the membrane softener Tween 80 allows the passage of the Transfersomes into the fungal cell, and (c) fungal lipases digest the invading Transfersome® vesicles thereby releasing their cytotoxic content. As this mode of action of Transfersomes is independent of the drug cargo, these results demonstrate the potential of Transfersomes in the treatment of all fungal diseases.

## Introduction

Targeted delivery of therapeutic drugs has the potential to reduce the effective drug dosage and avoid toxic side-effects, while maintaining the desired response [1]. One way to achieve this is through the use of lipid carrier vesicles [2], [3] that, when locally applied, penetrate the skin and allow targeted delivery of the enclosed drug. In recent years, numerous studies have reported the successful use of an ultra-deformable lipid vesicle, the Transfersome® [4]–[9]; overview in [10], [11]. The membrane of the Transfersome® consists of a lipid (e.g. phosphatidylcholine) and a membrane-softening agent (e.g. Tween 80). Due to its characteristic membrane composition, the Transfersome® can extremely deform, enabling spontaneous and efficient penetration of human skin by passing through intercellular spaces that are 5–10-times smaller than the size of the vesicle [1], [6]. This technology can efficiently deliver therapeutically active drugs across the skin barrier to subcutaneous tissue, and comparative studies have shown that Transfersome®-enclosed therapeutic drugs are more efficient than when applied in a conventional way (e.g. hydrogel application [4], [12]). This allows a more targeted and measured therapeutic approach that is based on a lower and, therefore, more tolerable drug dosage.

Onychomycosis is a common fungal disease of the nail, infecting up to 20% of the population over age 40 [13]. It is most frequently caused by the dermatophytes *Trichophyton rubrum* and *T. mentagrophytes* [14]. The treatment of onychomycosis has improved considerably since the introduction of the oral antifungal agents terbinafine and itraconazole [15]. However, drug–drug interactions and hepatotoxicity have been reported among patients treated with these oral antifungals [16], [17], highlighting the need for effective topical therapies which would avoid systemic exposure to the antifungal agent. *In vitro* experiments have demonstrated that TDT 067 (a topical formulation of 1.5% terbinafine in Transfersome®) has potent inhibitory activity against dermatophytes [18]. These experiments also demonstrated that TDT 067 has enhanced antifungal activity compared with “naked” terbinafine [18]. Following treatment with TDT 067, dermatophyte hyphae isolated from clinical samples showed extensive ultra-structural changes, indicative of death of the pathogen [19]. In both clinical samples and in *T. rubrum* hyphae exposed to TDT 067 *in vitro*, the fungal cells accumulated characteristic structures that appear solid and grayish in electron microscopy studies and that fill the dying pathogen [19], [20]. However, neither the nature of these structures, nor the precise mechanisms underlying the increased antifungal activity of TDT 067 is known.

This paper addresses the mode of action of TFVs in antifungal delivery. Terbinafine is known to inhibit a key fungal enzyme in sterol biosynthesis [21], [22]. As sterols determine membrane properties and organization of membrane sub-domains [23], the paper investigates the effects of terbinafine-loaded TFVs and conventional terbinafine on organelle organization and dynamics in the model fungus *Ustilago maydis*. It is shown here that entry of terbinafine-loaded TFVs into the fungal cell depends on the amount of the surfactant in their membrane and that they are more cytotoxic than the naked fungicide. This is due to an enzymatic and ATP-dependent digestion of the passively invading TFVs, which releases their content and increases the fungicide concentration locally. This degradation, as well as the cytotoxicity of terbinafine-loaded TFVs, can be neutralized by the addition of lipase inhibitors. This suggests that TFVs act as “Trojan Horses” that get degraded by fungal lipases, thereby releasing the fungicide. As this mode of action is not depending on the loaded drug, Transfersome technology promises an efficient way of treating fungal diseases in general.

## Results

### Delivery in Transfersome® vesicles enhances the antifungal activity of terbinafine

To test the efficiency of terbinafine-containing TFVs, a strain of *U. maydis*, in which organelles were stably labeled with specific fluorescent marker proteins, was incubated with various concentrations of terbinafine to enable the observation of the dynamic behavior and the organization of nuclei, peroxisomes, vacuoles, mitochondria, early endosomes, and the endoplasmic reticulum (for a description of fungal strains, see Methods and **Table 1**).

**Table 1:** Genotype of strains and plasmids used in this study.

AB33	*a2* P*narbW2* P*narbE1 ble* ^R^	[80]
AB33GLAct	*a2* P*narbW2* P*narbE1 ble* ^R^/poGLifeAct	[39]
AB33G_3_Dyn2	*a2* P*nar-bW2* P*nar-bE1*, P*dyn2-3xegfp-dyn2, ble* ^R^, *hyg* ^R^	[79]
AB33GRab5a	*a2* P*narbW2* P*narbE1, ble* ^R^/poGRab5a	[79]
AB33ERG	*a2* P*narbW2* P*narbE1, ble* ^R^/pERGFP	[24]
AB33GSKL	*a2* P*narbW2* P*narbE1, ble* ^R^/poGSKL	[42]
AB33LgaG	*a2* P*narbW2* P*narbE1, ble* ^R^/poLgaG	[42]
AB33CpYG	*a2* P*narbW2* P*narbE1, ble* ^R^/poCpyG	This study
AB33nG	*a2* P*narbW2* P*narbE1, ble* ^R^/pnGFP	This study
poGLifeAct	P*otef-egfp-ABP140^1-17_modified^ cbx* ^R^	[39]
poGRab5a	P*otef-egfp-rab5a, nat* ^R^	[30]
pERGFP	P*otef-cal^s^-gfp*-HDEL, *cbx* ^R^	[24]
poGSKL	P*otef-egfp*-SKL, *cbx* ^R^	[42]
poLgaG	P*otef-lga-egfp, cbx* ^R^	[42]
poCpYG	P*otef-cpY-gfp, nat* ^R^	This study
pnGFP	*gal4(s):egfp, cbx*	[78]

a, b, mating type loci; P, promoter; -, fusion; hygR, hygromycin resistance; bleR, phleomycin resistance; natR, nourseothricin resistance; cbxR, carboxin resistance/, ectopically integrated; otef, constitutive promoter; nar, conditional nitrate reductase promoter; E1, W2, genes of the b mating type locus; LifeAct, first 17 aa of Abp140 dyn2: C-terminal half of the dynein heavy chain; rab5a, small endosomal Rab5-like GTPase; cpy, carboxy-peptidase Y; calS, signal sequence of calreticulin from rabbit, HDEL, endoplasmic reticulum retention signal; gal4s, nuclear localization signal of the GAL-4 DNA binding domain from pC-ACT1 (Clontech); SKL, peroxisomal targeting signal; lga2, putative mitochondrial matrix protein.

**Figure 1:**
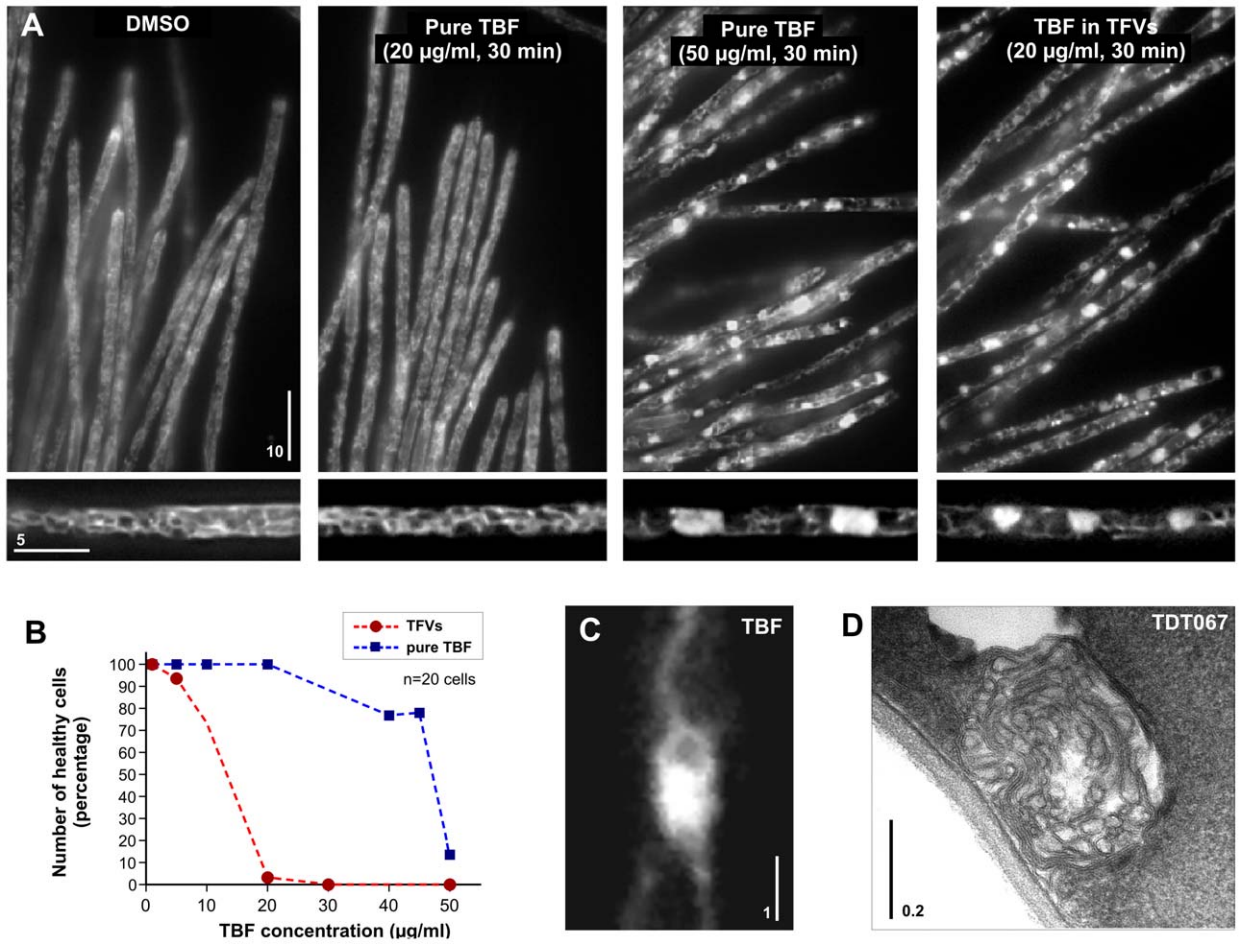
The effect of terbinafine and terbinafine-loaded fluorescent TFVs on the endoplasmic reticulum. (**A**) The organization of the endoplasmic reticulum (labeled with a green fluorescent protein fused to an ER-retention signal and an ER targeting sequence [24]; see Methods) in hyphae treated with the solvent dimethyl sulfoxide (DMSO), terbinafine encapsulated in fluorescent TFVs (TBF in TFVs), and pure terbinafine (pure TBF). Globular endoplasmic reticulum structures were formed after 30-min treatment with 20 µg/ml terbinafine applied in Transfersome® suspension (see also **Movie S1**). Similar structures occurred when cells were treated with 50 µg/ml terbinafine. Bar represents micrometers. (**B**) Graph showing the proportion of cells with normally organized endoplasmic reticulum in cells treated with terbinafine or terbinafine-loaded TFVs (TFVs). (**C**) Detail of a globular endoplasmic reticulum structure after treatment with 50 µg/ml terbinafine. The ER structure was labeled with a green fluorescent protein fused to an ER-retention signal and an ER targeting sequence [24]. Bar represents a micrometer. (**D**) Electron micrograph of globular membrane accumulation in TFVs-treated cells. Bar represents a micrometer.

In order to recognize specific effects of terbinafine, we focused on changes in organelle appearance after 30 min of exposure. Under these conditions, terbinafine treatment had no obvious effect on the organization of most organelles (**Fig. S1**; for description of all GFP-based organelle markers see Methods). A minor effect was observed for mitochondria, which showed a tendency to undergo fragmentation (**Fig. S1**). However, terbinafine did induce a dramatic reorganization of the endoplasmic reticulum. Control cells treated with the solvent dimethyl sulfoxide (DMSO; **Fig. 1A**, **S1**) showed a peripheral network of endoplasmic reticulum tubules, which corresponded well with previous reports in untreated cells [24]. Treatment of cells with 50 µg/ml terbinafine led to the formation of globular endoplasmic reticulum structures (**Fig. 1A**, 50 µg/ml TBF; **Fig. 1B, 1C**). Globular, endoplasmic reticulum structures were also induced by treatment of cells with terbinafine-loaded TFVs, but the effect occurred at much lower concentrations of terbinafine (**Fig. 1A**, 20 µg/ml, TBF in TFVs; **Fig. 1B**; **Movie S1**). Conventional terbinafine had no obvious effect at this concentration (**Fig. 1A**, 20 µg/ml, TBF; **Fig. 1B**). The globular endoplasmic reticulum structures appeared as membrane aggregations under electron microscopy (**Fig. 1D**), suggesting that terbinafine interferes with the membrane organization of the endoplasmic reticulum.

### Transfersome® vesicles penetrate the fungal cell

**Figure 2:**
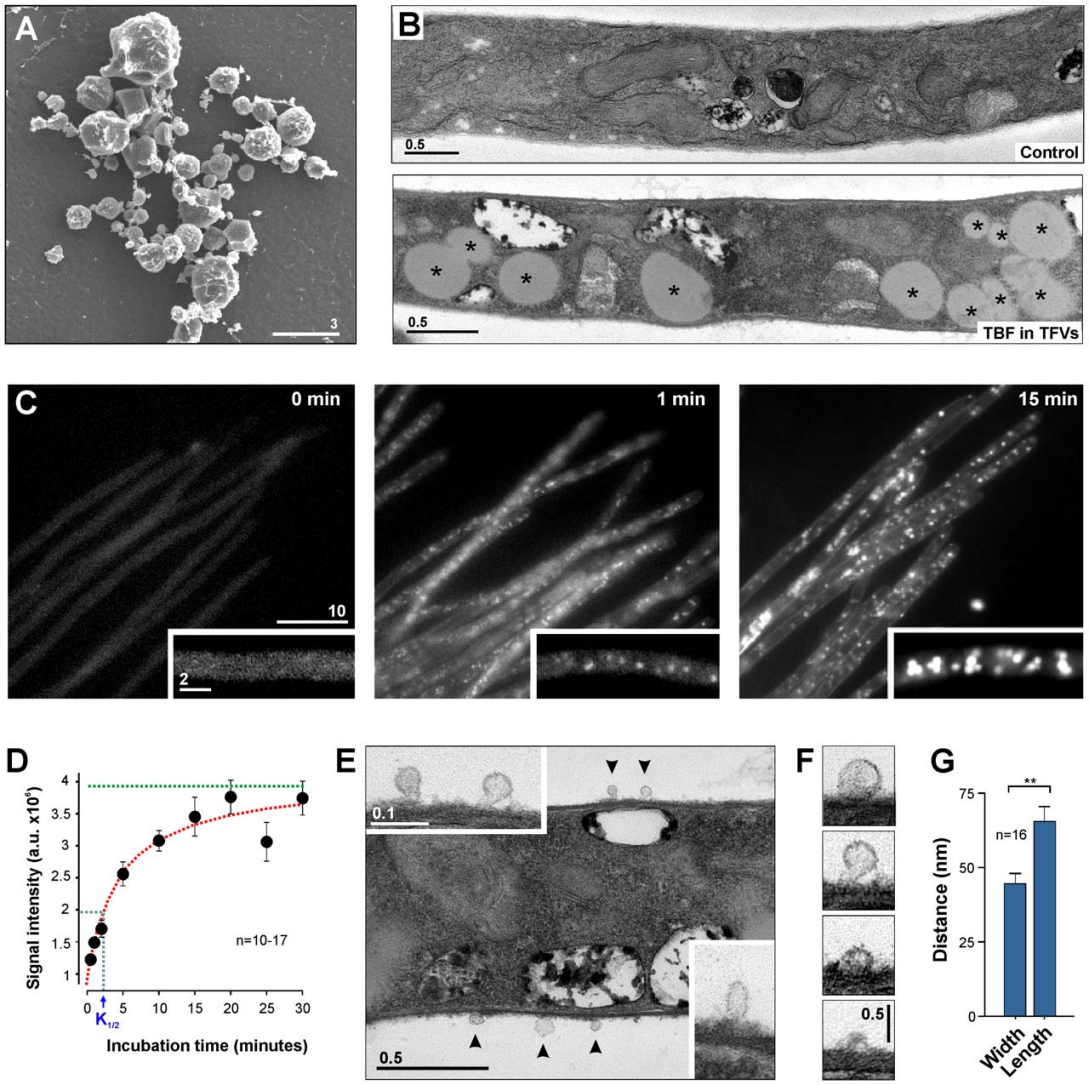
Entry of terbinafine-loaded TFVs into the fungal cell. (**A**) Scanning electron micrograph of terbinafine-loaded Transfersome® vesicles. Bar represents micrometers. (**B**) Electron micrographs of an untreated hyphal cell (control) and after 30 min of incubation with fluorescent terbinafine-loaded TFVs (TBF in TFVs). The cell accumulates large droplets (asterisk). Bar represents micrometers. (**C**) Uptake of fluorescently labeled terbinafine-loaded Transfersome® vesicles. Prior to Transfersome® treatment, the cells show weak background fluorescence (0 min). Small and relatively faint fluorescent signals appear ∼1 min after adding fluorescent TFVs (1 min) and strong fluorescence is detected after ∼15 min of incubation. See also **Movie S2**. Bars represent micrometers. (**D**) Graph showing the kinetics of Transfersome® uptake. Each data point represents the mean ± standard error of the mean of 10–17 measurements. Note that uptake is rapid (K½ ∼2 min) and that uptake reaches a maximum (green dotted line). (**E**) Electron micrograph showing vesicles (arrowheads) attached to a hyphal cell treated with fluorescent terbinafine-loaded TFVs. Note that the attached vesicles are elongated (right lower corner). Bars represent micrometers. (**F**) Image series of electron micrographs of TFVs at different stages of entry. Note the deformation of the vesicles. Bar represents micrometers. (**G**) Bar chart showing the length and the width of vesicles attached to the outer fungal cell wall. Note that only those structures were measured that showed a lumen (e.g. the upper two vesicles in the image series given in F). Mean ± standard error of the mean of 16 measurements. Statistical significance difference at *P*<0.001 is indicated by double asterisks.

To investigate whether the TFVs can enter the fungal cell, TDT 067 was used that was labeled with 1,6-diphenyl-1,3,5-hexatriene, a dye that specifically labels lipid membranes [25]. Scanning electron microscopy of these fluorescent terbinafine-loaded TFVs indicated that the applied suspension contained vesicles of various sizes (**Fig. 2A**). This size variation was also found when non-fluorescent TFVs were visualized in scanning electron microscopy (not shown). However, using photon correlation spectroscopy TFVs were found to be of uniform size, with an average diameter of ∼90 nm and a size distribution width of 15 nm (Dr. U. Vierl, C.P.M. ContractPharma GmbH & Co. KG, Feldkirchen-Westerham, Germany, pers. communication), suggesting that the size variation observed in my experiments are most likely due to dehydration occurring during the preparation process.

When applied to *U. maydis* hyphae, the fluorescent terbinafine-loaded TFVs induced the formation of characteristic structures within 15–30 min (**Fig. 2B**; “Control” indicates untreated cells; “TFVs” indicates Transfersome® treatment for 30 minutes; new structures indicated by asterisks). These structures were globular and strongly fluorescent (**Fig. 2C**, 15 min; **Movie S2**), demonstrating that they were derived from uptake of fluorescently labeled terbinafine-loaded TFVs. The same phenotype was observed when terbinafine-free fluorescent TFVs were applied (not shown). The kinetics of this uptake was investigated by measuring the average fluorescent intensity along several micrometers in terbinafine-loaded Transfersome®-treated hyphae. Almost no fluorescent signal was detected before the terbinafine-loaded Transfersome® suspension was applied (**Fig. 2C**, 0 min; **Fig. 2D**); however, spots of fluorescence appeared shortly after incubation (**Fig. 2C**, 1 min; **Fig. 2D**) and strongly labeled globular structures were observed after 15–30 min of incubation (**Fig. 2C**, 15 min; **Fig. 2D**). Interestingly, the increase in fluorescent intensity reached a maximum value after 15–20 min (**Fig. 2D**, green dotted line), suggesting that the cell has a maximum capacity for uptake or cellular presence of the fluorescent terbinafine-loaded TFVs.

Next we set out to capture the moment of uptake by fixing cells shortly after applying TFVs was addressed. Transmission electron microscopy revealed small vesicles of about 50–80 nm attached to the outer fungal cell wall (**Fig. 2E**, insets and arrowheads; **Fig. 2F**), which most likely are TFVs that were fixed during invasion of the fungal cell. It was observed that these vesicles were usually elongated (**Fig. 2E**, inset in the lower right corner; **Fig. 2G**) and occasionally deformed (**Fig. 2F**, arrowhead). This corresponds well with the previously described capacity of TFVs to undergo extreme curvature [5], [6].

### The entry of the Transfersome® into fungal cells is dependent on the concentration of Tween 80 in the vesicle membrane

**Figure 3:**
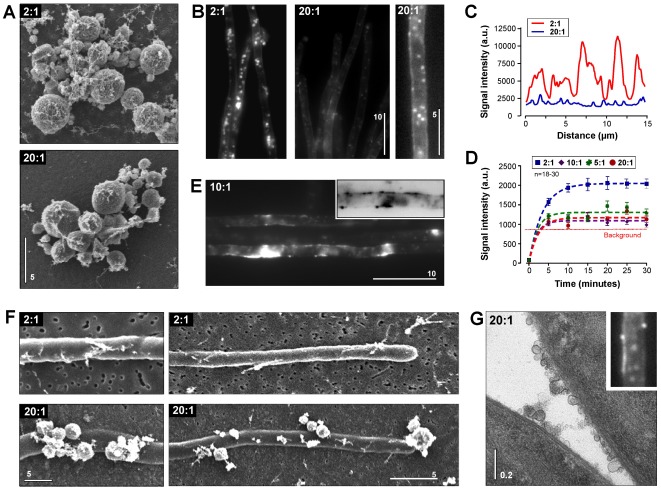
The effect of Tween 80 concentrations on Transfersome® uptake. (**A**) Scanning electron micrographs showing fluorescent terbinafine-loaded TFVs with the standard phosphatidylcholine to Tween 80 ratio (2∶1) and at 10-times reduced Tween 80 concentration (20∶1). Both vesicle carriers show a similar appearance. Bar represents micrometers. (**B**) Cellular fluorescence of Transfersome®-derived lipids after 30 min of incubation with standard fluorescent terbinafine-loaded fluorescent TFVs (2∶1) and fluorescent terbinafine-loaded fluorescent TFVs with a 10-times lower concentration of Tween 80 (20∶1). Treatment with the latter resulted in very little cellular fluorescence and only a few faint signals were detected (right panel). Note that image “right panel, 2∶1” and “middle panel, 20∶1” were identically scaled and processed using MetaMorph. The image “20∶1, left panel” was contrast and brightness processed and gamma-values were adjusted to visualize the faint Transfersome®-derived fluorescent signals. Bar represents micrometers. (**C**) Intensity profile of fluorescent signals in hyphae treated with standard fluorescent terbinafine-loaded TFVs (2∶1) and fluorescent terbinafine-loaded TFVs with a 10-times lower concentration of Tween 80 (20∶1). (**D**) Graph showing the uptake kinetics of fluorescent TFVs with various ratios of Tween 80 to phosphatidylcholine (2∶1, 5∶1, 10∶1, 20∶1). Uptake of TFVs depends on a high concentration of Tween 80 in their membrane. (**E**) Fluorescent patches at the periphery of hyphal cells treated with fluorescent vesicles at a 10∶1 ratio of Tween 80 to phosphatidylcholine. Inset shows a contrast inverted image. Note that similar patches were found in experiments using 5∶1 and 20∶1 phosphatidylcholine to Tween 80 ratios. Bar represents micrometers. (**F**) Scanning electron micrographs showing hyphal cells treated with fluorescent terbinafine-loaded TFVs at a 2∶1 and 20∶1 ratio of Tween 80 to phosphatidylcholine. Vesicles attach to the hyphal surface at a lower concentration of Tween 80, suggesting that Tween 80 reduces unspecific binding of Transfersome® membranes to the cell wall. Bars represent micrometers. (**G**) Transmission electron micrograph of hyphal cells treated with fluorescent terbinafine-loaded TFVs at a 20∶1 ratio of Tween 80 to phosphatidylcholine. Numerous small vesicles are attached to the cell wall. Inset shows a uniform fluorescent rim at the surface of a cell which most likely corresponds to the lawn of attached vesicles shown in the electron micrograph. Bars represent micrometers.

To test for the importance of the concentration of Tween 80 in the Transfersome®, fluorescently labeled Transfersome® suspensions were generated that varied in the ratio of phosphatidylcholine to Tween 80, ranging from 2∶1 (the standard ratio used in most experiments) to 20∶1 (provided by Dr. U. Vierl, C.P.M. ContractPharma GmbH & Co. KG, Feldkirchen-Westerham, Germany). Control experiments demonstrated that the variation in Tween 80 concentration did not affect the size and shape of the vesicles when prepared for scanning electron microscopy (**Fig. 3A**; phosphatidylcholine to Tween 80 ratio indicated by “2∶1” and “20∶1”). When applied to fungal hyphae, 2∶1 terbinafine-loaded TFVs rapidly appeared in the fungal cells and accumulated in the described globular fluorescent structures (**Fig. 3B**, 2∶1, left panel). In contrast, low concentration of Tween 80 prevented uptake of the fluorescent vesicles (**Fig. 3B**, 20∶1, middle panel) and only few faint signals were observed (**Fig. 3B**, 20∶1, right panel; note that image “right panel 2∶1” and “middle panel 20∶1” were identically scaled and processed; the image “20∶1 left panel” was contrast and brightness processed and gamma-values were adjusted to visualize the faint fluorescent signals). The difference in appearance of fluorescent signals is most obvious in comparative line scan analysis of the fluorescent intensities that was done on unprocessed images (**Fig. 3C**).

Next the uptake kinetics of fluorescent terbinafine-loaded TFVs at phosphatidylcholine to Tween 80 ratios of 2∶1, 5∶1, 10∶1, and 20∶1 was investigated. Again, it was found that the standard 2∶1 TFVs were rapidly taken up and reached a plateau of maximal fluorescence intensity after 15–20 min (**Fig. 3D**, 2∶1). In contrast, very little fluorescence appeared when the concentration of Tween 80 in the Transfersome® membrane was reduced (**Fig. 3D**, 5∶1, 10∶1, 20∶1), suggesting that these TFVs were not able to enter the fungal cell. Indeed, no globular structures were observed in electron micrographs of hyphae treated with 20∶1 fluorescent terbinafine-loaded TFVs (not shown). These results indicate that high concentrations of Tween 80 are required to enable the Transfersome® to enter fungal cells. This is in contrast to Transfersomes passing through the skin where lower concentrations are sufficient to take the vesicles through and the operating range is wider.

We noted that hyphal cells treated with low-Tween 80 TFVs often showed sheet-like fluorescent patches (**Fig. 3E**). These fluorescent patches appeared at the periphery of the cell (**Fig. 3E**, inset, contrast was inverted for better visualization), suggesting that they represent TFVs that were attached to the fungal cell wall but did not enter the cell. This possibility was tested using scanning electron microscopy. Hyphal cells that were treated with the standard 2∶1 terbinafine-loaded TFVs showed few attached structures which were too small to be resolved as vesicles (**Fig. 3F**). In contrast, a reduced Tween 80 concentration enabled attachment of the vesicles to the fungal cell wall (**Fig. 3F**). At a higher magnification in transmission electron microscopy, large patches of vesicles were observed attached to the fungal cell wall (**Fig. 3G**, 20∶1) that matched the peripheral patches of fluorescence (**Fig. 3G**, inset). These data suggested that Tween 80 is required to overcome the fungal cell wall. This conclusion is supported by the observation that 20∶1 TFVs are able to enter cell-wall free protoplasts, although significantly less fluorescence was measured inside protoplasts when compared to the standard 2∶1 TFVs (*P*=0.0008, two-tailed t-test; **Fig. S2**, 2∶1 and 20∶1). Taken together, these data indicate that the level of Tween 80 is important for the activity of the Transfersome® against the fungus as it allows hyper-deformation of the vesicle membrane which is required for it to pass through the small holes in the fungal cell wall. In addition, Tween 80 appears to reduce unspecific interaction of the vesicles with the fungal cell wall, which might hinder the entry into the cell.

### Entry of terbinafine-loaded Transfersome® vesicles into the fungal cell is a passive process

**Figure 4:**
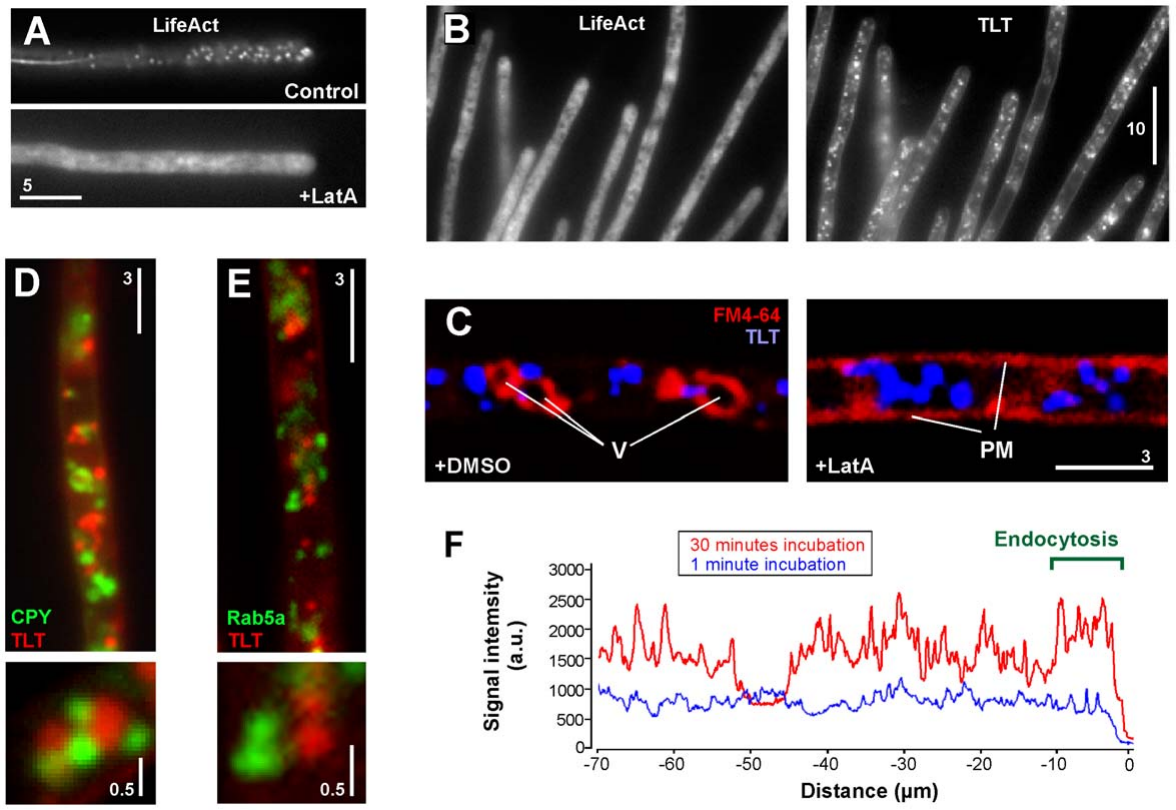
Evidence against endocytic uptake of Transfersome® vesicles. (**A**) A hyphal cell showing F-actin structures labeled with lifeAct-GFP in the presence of the solvent DMSO (control) and in the presence of 10 µM of the actin inhibitor latrunculin A (LatA). Note that latrunculin A disrupts endocytic F-actin patches and F-actin cables. Bar represents micrometers. (**B**) Uptake of fluorescently labeled terbinafine-loaded TFVs (TLT) after 30 min of incubation in the absence of F-actin patches (background fluorescence is due to unbound GFP-LifeAct protein [35]–[37]). Note that uptake occurs at normal rates, demonstrating that F-actin is dispensable for entry of the vesicles into the fungal cell. Bar represents micrometers. (**C**) Co-localization of fluorescent terbinafine-loaded TFVs (blue, TLT) and the endocytic marker dye FM-64 (red) in the presence of the solvent dimethyl sulfoxide (DMSO) or Latrunculin A (LatA). In control cells the marker dye FM4-64 concentrates in vacuoles (V), whereas disruption of the F-actin cytoskeleton abolishes endocytic uptake and the dye remains in the plasma membrane (PM). In both cases, TFVs accumulate in the cytosol. Bar represents micrometers, (**D**) Localization of fluorescent terbinafine-loaded TFVs (red, TLT) and carboxypeptidase Y (green, CPY), a marker for vacuoles. Note that both structures do not co-localize. Bar represents micrometers. (**E**) Localization of fluorescent terbinafine-loaded TFVs (red, TLT) and the small GTPase Rab5a (green, Rab5a), a marker for early endosomes. Note that both structures do not co-localize. Bar represents micrometers. (**F**) Graph showing fluorescent intensity scans along the hyphal cell at 1 min and 30 min after treatment with fluorescent terbinafine-loaded TFVs. Note that endocytosis is expected to occur at the hyphal tip only (indicated by “Endocytosis”), whereas fluorescent signals occur along the entire cell.

It was reported that whole vesicles can cross the fungal cell wall [26], [27], which raises the possibility that Transfersome vesicles enter the fungal cell via the endocytic pathway. Uptake of material into the fungal cell by endocytosis has been shown to occur in various filamentous fungi [28]–[31], including *U. maydis* [32], [33]. The process of endocytosis is dependent on F-actin patches that mark the region of endocytic uptake [34]. In fungi, these patches can be visualized using a green fluorescent protein (GFP)-based probe lifeAct-GFP [35]–[37]; overview in [38]. In *U. maydis*, lifeAct-GFP labels cables and endocytic patches (**Fig. 4A**, upper panel; [39]). The use of 10 µM latrunculin A (a specific inhibitor of F-actin [40]) destroyed these F-actin structures in *U. maydis* (**Fig. 4A**, lower panel; [41]), which is predicted to block endocytosis. However, in latrunculin A-treated hyphae, terbinafine-loaded fluorescent TFVs were still taken up into the fungal cells (**Fig. 4B**). To confirm that endocytosis was blocked under these conditions, co-staining of TFVs with the lipophilic endocytic marker dye FM4-64 was done. Indeed, disruption of F-actin by latrunculin A abolished the concentration of FM4-64 in the vacuole membrane and instead the dye remained in the plasma membrane (**Fig. 4C**, PM). In contrast, the dye accumulates in vacuoles when cells were treated with the solvent dimethyl sulfoxide (DMSO; **Fig. 4C**, V). This result confirms that F-actin is required for endocytic uptake of FM4-64 into the fungal cell. However, fluorescent Transfersome®-membranes accumulate in the cytoplasm of latrunculin A -treated, again indicating the Transfersome® uptake occurs independently of the endocytic pathway.

To determine if terbinafine-loaded TFVs reach the fungal vacuole, where the content of endocytic vesicles is known to be deposited [33], we co-localized Transfersome®-derived fluorescent signals with the early endosome marker GFP-Rab5a [32] and with a vacuolar marker carboxy-peptidase Y-GFP [42]. We did not observe co-localization of fluorescent terbinafine-loaded TFVs and early endosomes or vacuoles, respectively (**Fig. 4D, 4E**; **Fig. S3**). This also argues against an endocytic uptake of Transfersome® vesicles.

Finally, the site of uptake of fluorescent terbinafine-loaded TFVs into the fungal hyphal cell was monitored. As recent work has demonstrated that endocytosis occurs predominantly near the growing cell tip [29], we expected high fluorescence near the hyphal tip, which would indicate that the TFVs enter the cell via endocytosis. To test this, we incubated hyphal cells for 1 min and 30 min and performed an intensity scan along the length of the hyphal cell. We found that fluorescent signals appeared along the entire cell shortly after adding the TFVs and no concentration was detected near the hyphal tip (**Fig. 4F**). This strengthens our argument against a role for endocytosis in Transfersome® uptake.

### Transfersome® membranes form lipid droplets

**Figure 5:**
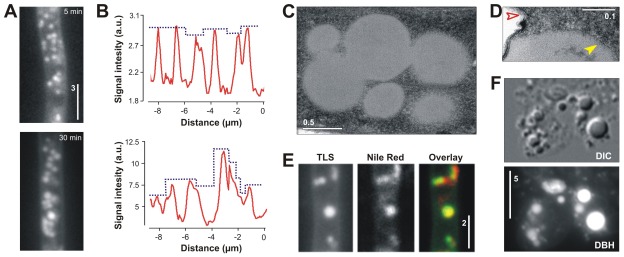
Characterization of fluorescently labeled Transfersome®-derived structures. (**A**) Images showing hyphal cells that were incubated for 5 min (upper panel) and 30 min (lower panel) with fluorescent terbinafine-loaded Transfersome® vesicles. Note that at early stages fluorescent signals are small and even in intensity, whereas signals become larger and differ in intensity with time. Note also that the difference in intensity between the upper and lower panel are not representative due to image processing (for comparison of intensities see panel B). Bar represents micrometers. (**B**) Graphs showing fluorescent intensity scans along the hyphal cells treated with fluorescent terbinafine-loaded TFVs for 5 min (upper graph) and 30 min (lower graph). At 5 min, the signals are faint (∼3000 arbitrary units) and even in intensity (blue dotted line), whereas signal intensity increases with time (lower graph; up to ∼12000 arbitrary units) and becomes unevenly distributed (blue dotted line). (**C**) Electron micrograph showing intracellular structures after treating cells with fluorescent terbinafine-loaded Transfersome® vesicles for 30 min. Bar represents micrometers. (**D**) Electron micrograph showing a vacuole and a Transfersome®-derived structure. The vacuole is surrounded by a double membrane (red arrowhead), whereas the Transfersome®-derived structure -derived structure is surrounded by a single membrane, suggesting that it represents a lipid droplet. Bar represents micrometers. (**E**) Co-localization of fluorescent structures (TLS) and the lipid marker dye Nile Red (Nile Red). Bar represents micrometers. (**F**) Lipid structures derived from osmotically disrupted protoplasts of fungal cells treated with fluorescent TFVs for 30 min. After breaking the wall-less cells, structure of high contrast remain (upper panel, DIC) that show strong 1,6-diphenyl-1,3,5-hexatriene fluorescence (lower panel, DBH). The high retractile index of the globular structures indicates that the fluorescent structures are lipid droplets. Bar represents micrometers.

Treatment with fluorescent terbinafine-loaded TFVs results in the appearance of cytosolic fluorescent signals, suggesting that the vesicles enter the fungal cell. The first signals appeared a few minutes after TFVs application, and were relatively faint and of even intensity (**Fig. 5A**, upper panel; **Fig. 5B**, upper graph). With time, these signals unevenly increased in sensitivity (**Fig. 5A**, lower panel; **Fig. 5B**, lower graph; see also above **Fig. 4F**). The strongest globular signals had diameters of up to 700 nm and appeared solid in electron micrographs (**Fig. 5C**; see above **Fig. 2B**). This suggests that they are not accumulations of hollow and intact TFVs. Electron micrographs also demonstrated that the fluorescent terbinafine-loaded Transfersome®-induced globular structures are surrounded by a single membrane (**Fig. 5D**; solid arrowhead; compare with double membrane of a vacuole indicated by open arrowhead). This is characteristic of lipid droplets [43], raising the possibility that these structures are lipid bodies that derived from fused Transfersome® membranes. To test the nature of the globular bodies, we stained terbinafine-loaded Transfersome®-treated hyphal cells with the lipid dye Nile Red [44]. Indeed, we found a clear co-localization of the Nile Red with the fluorescent Transfersome®-derived structures (**Fig. 5E**). This suggests that these bodies are indeed lipid droplets. To gain further support for this conclusion, we generated cell-wall less protoplasts from fluorescent terbinafine-loaded Transfersome®-treated cells and disrupted them by osmotic shock. Structures of high retractile index were observed that showed a strong 1,6-diphenyl-1,3,5-hexatriene fluorescence (**Fig. 5F**). The optical features of the fluorescent droplets confirmed the notion that these fluorescent globular bodies are indeed lipid bodies.

### Transfersome®-derived lipid bodies are enzymatically degraded by lipases

**Figure 6:**
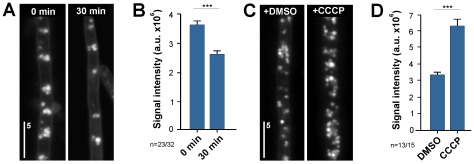
Evidence for an ATP-dependent degradation of fluorescent terbinafine-loaded TFVs. (**A**) Images showing hyphal cells that were incubated for 30 min with fluorescent terbinafine-loaded TFVs, washed twice and immediately observed (0 min) or incubated for an additional 30 min at room temperature (30 min). Note that the cellular fluorescence decreases with time. Bar represents micrometers. (**B**) Bar chart showing average signal intensities in hyphal cells that were incubated for 30 min with fluorescent terbinafine-loaded TFVs, washed twice and immediately observed (0 min) or incubated for an additional 30 min at room temperature (30 min). Bars represent mean ± standard error of the mean of 23 (0 min) and 32 (30 min) measurements. Statistical significance difference at *P*<0.0001 is indicated by triple asterisks. (**C**) Images showing hyphal cells that were incubated for 30 min with fluorescent terbinafine-loaded TFVs in the presence of the solvent DMSO and 100 µg/ml of the ionophore CCCP, which depletes cellular ATP levels. Bar represents micrometers. (**D**) Images showing hyphal cells that were incubated for 30 min with fluorescent terbinafine-loaded TFVs in the presence of the solvent DMSO and 100 µg/ml of the ionophore CCCP. Bars represent mean ± standard error of the mean of 13 (DMSO) and 15 (CCCP) measurements. Statistical significance difference at *P*<0.0001 is indicated by triple asterisks.

The appearance of lipid droplets suggested that TFVs were degraded after entry into the cell. This notion was supported by uptake kinetics that demonstrated a maximum plateau after 15 min (see above), suggesting that the lipid droplets degradation balances the intake. To gain experimental support for this conclusion, a pulse-chase experiment was performed. For this fungal cells were incubated for 20 minutes with Terbinafine-loaded fluorescent TFVs, the amount of cytosolic fluorescence was measured and the cells were transferred into Transfersome®-free medium for an additional 30 minutes. In these experiments, the number of Transfersome®-derived lipid bodies was significantly reduced (**Fig. 6A**) and that the associated fluorescence dropped (**Fig. 6B**). These results suggested that the cellular capacity for TFVs is determined by the activity of lipid-degrading enzymes. This was further tested by incubating hyphae with fluorescent terbinafine-loaded TFVs in the presence of the ionophore cyanide 3-chlorophenyl-hydrazone (CCCP). This inhibitor reversibly blocks cell respiration, resulting in reduced ATP levels [45]. The effect on *U. maydis* was blocking motility of fluorescently labeled dynein motors. In the presence of the solvent DMSO, dynein motors were rapidly moving along microtubules, which is known to be an ATP-dependent process [46]. In the presence of 100 µg/ml CCCP, all motility stopped within 30–45 min (**Movie S3**; **Fig. S4**). This confirmed that treatment with the ionophore led to a depletion of cellular ATP levels. When cells were incubated with CCCP or DMSO and fluorescent terbinafine-loaded TFVs, fluorescent signals were detected inside the fungal cells (**Fig. 6C**), again arguing that Transfersome® uptake is a passive process. However, ATP-depleted cells showed significantly stronger fluorescent signals than control cells (**Fig. 6D**), which is consistent with the notion that the terbinafine-loaded TFVs are a substrate for ATP-dependent metabolic processes inside the fungal cytoplasm. In summary, these experiments indicate that TFVs and lipid bodies are substrates for ATP-dependent enzymatic degradation.

### A lipase inhibitor prevents degradation and neutralizes the antifungal activity of terbinafine-loaded Transfersome®

**Figure 7:**
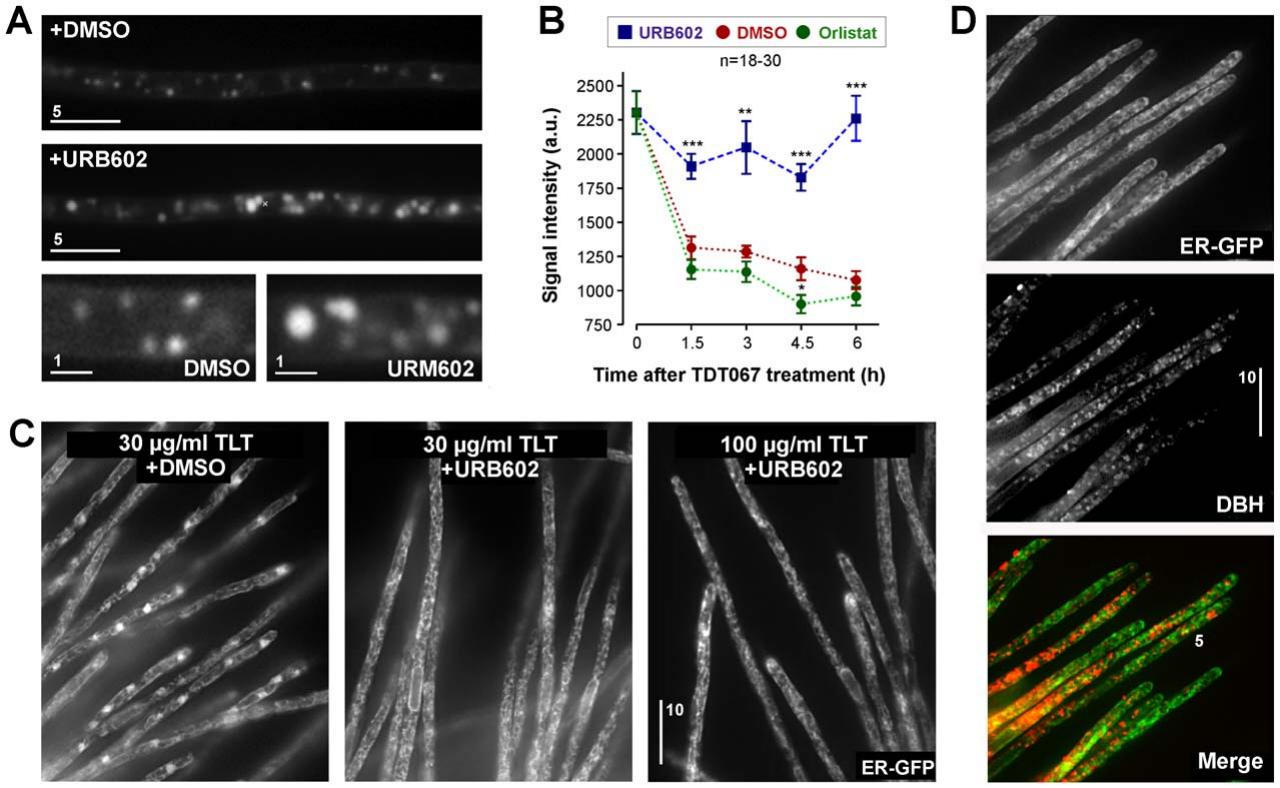
Inhibition of terbinafine-loaded Transfersome® degradation and cytotoxicity by lipase inhibitors. (**A**) Images showing hyphal cells treated with fluorescent terbinafine-loaded TFVs for 30 min, followed by incubation in Transfersome®-free conditions in the presence of the solvent DMSO (+DMSO) and the lipase inhibitor URB602. Bar represents micrometers. (**B**) Graph showing average fluorescent intensity in hyphal cells treated with fluorescent terbinafine-loaded TFVs for 30 min, followed by incubation in Transfersome®-free conditions in the presence of the solvent DMSO (+DMSO) and the lipase inhibitors Orlistat and URB602. Bars represent mean ± standard error of the mean of 18–30 measurements per data point. Statistical significance difference at *P*<0.001 is indicated by double asterisks; triple asterisks indicate difference at *P*<0.0001. (**C**) Images showing the defect in organization of the endoplasmic reticulum in the presence of 30 µg/ml terbinafine encapsulated in fluorescent TFVs (TLT) and the solvent DMSO, 30 µg/ml terbinafine encapsulated in fluorescent TFVs and 50 µM of the lipase inhibitor URB602, and 100 µg/ml terbinafine encapsulated in fluorescent TFVs and 50 µM of the lipase inhibitor URB602. Note that the lipase inhibitor prevented the induction of collapsed endoplasmic reticulum structures (see above **Fig. 1**) even at high Transfersome® concentrations. Bar represents micrometers. (**D**) Fungal cells treated with fluorescent TFVs for 30 min in the presence of 50 µM of the lipase inhibitor URB602. Small fluorescent signals appear inside the hyphal cells but no collapsed endoplasmic reticulum structures are visible, indicating that the terbinafine-loaded vesicles enter the cell, but the presence of the lipase inhibitor prevents release of the drug and thereby inhibits the cytotoxicity of the Transfersome® carriers. Bar represents micrometers.

The ATP dependency of the degradation of Transfersome®-derived lipids suggested some metabolic activity of fungal lipases [47], [48]. We screened the published genome of *U. maydis* (http://mips.gsf.de/genre/proj/ustilago/) and identified 21 potential lipases. Nine of these contain a secretion signal and might act outside the cell (predicted by http://www.cbs.dtu.dk/services/TargetP/); two are predicted to locate within mitochondria; and one lipase contains a PX-like domain (predicted by http://www.ebi.ac.uk/interpro/), suggesting that it binds to the lipids of early endosomes [49]. The remaining nine lipases could be cytosolic and have the potential to act on the Transfersome® membranes. To test this lipase activity was inhibited by specific inhibitors and the effect on Transfersome®-degradation was monitored. Cells were incubated with fluorescent TFVs, followed by extended incubation in Transfersome® free medium that was supplemented with the solvent DMSO and the specific lipase inhibitors Orlistat [50] and URB602 [51], [52]. The degradation of fluorescent terbinafine-loaded Transfersome®-derived lipids occurred in DMSO and in the presence of the gastric and pancreatic lipase inhibitor Orlistat, but was strongly inhibited when lipases were inhibited by a monoacylglycerol inhibitor URB602 (**Fig. 7A, 7B**). This result added further support to the notion that terbinafine-loaded TFVs are a substrate for cytosolic lipid-degrading enzymes.

On the basis of these results, we investigated whether attack by lipase activity is responsible for the release of terbinafine from the TFVs. To test this we monitored the antifungal activity of fluorescent terbinafine-loaded TFVs in the presence of the lipase inhibitor URB602. Treatment with 30 µg/ml terbinafine in TFVs led to collapse of endoplasmic reticulum membrane structures in almost all cells (**Fig. 7C**; see also **Fig. 1B**). However, in the presence of 50 µM URB602, terbinafine-loaded Transfersome® suspension had no effect on the endoplasmic reticulum organization (**Fig. 7C**). Even treatment with 100 µg/ml terbinafine in TFVs only led to minor disruption of the endoplasmic reticulum organization when lipases were inhibited (**Fig. 7C**, right panel). This was not due to a defect in cellular entry of the fluorescent terbinafine-loaded TFVs, as fluorescent signals were found within the fungal cells (**Fig. 7D**). These data add support to the conclusion that TFVs are digested by cytosolic fungal lipases. This activity appears to release the enclosed antifungal within the fungal cell.

## Discussion

### Terbinafine affects the organization of the fungal endoplasmic reticulum

Terbinafine is a well-characterized drug that is known to inhibit squalene epoxidase, a key enzyme in the ergosterol and cholesterol biosynthetic pathways [21], [22]. It has been reported that terbinafine is particularly potent against fungal enzymes, but has only minor activity against squalene epoxidase from rat liver [53]. Therefore, terbinafine is a valuable antifungal agent that is particularly effective in the treatment of dermatophyte onychomycosis [15], [54]. Fungal-specific ergosterol is found in the plasma membrane where it is involved in numerous processes, including endocytosis [55], and cell–cell fusion during mating [56]. However, it also resides in other organelles (e.g. mitochondria, vacuoles/lysosome, endoplasmic reticulum), where it might support other essential and more unexpected functions (e.g. maintaining pH homeostasis at the vacuolar membrane [57]). As part of the fungal membrane, ergosterol determines membrane fluidity and sub-domain organization (e.g. formation of lipid rafts [58], [59]).

In our experiments, terbinafine caused a collapse of the endoplasmic reticulum in *U. maydis*. In the budding yeast *S. cerevisiae*, inhibition of ergosterol synthesis causes the influx of calcium into the endoplasmic reticulum [60], which raises the possibility that the observed collapse of the endoplasmic reticulum could be an indirect consequence of a change in ion homeostasis. Altered calcium homeostasis resulted in alteration of the fungal microtubule cytoskeleton [61], [62], but no effect on the organization of the endoplasmic reticulum was reported. Thus, it seems unlikely that changes in calcium levels caused the loss of endoplasmic reticulum organization we observed in our study. Alternatively, it is possible that inhibition of ergosterol synthesis by terbinafine alters the membrane properties of this compartment, thereby causing the observed collapse of the endoplasmic reticulum tubules. Ergosterol biosynthesis has been shown to be involved in mitochondria and vacuole organization [63], [64], illustrating that those membrane lipids can play crucial roles in maintaining the morphology of organelles. The endoplasmic reticulum is known to consist of sub-domains and its sub-cellular organization is essential for its cellular function [65]. Thus, the terbinafine-induced collapse of the endoplasmic reticulum network is predicted to interfere with its essential function, which might account in part for the antifungal activity of terbinafine.

### Entry of terbinafine-loaded Transfersome® vesicles into the fungal cell requires the surfactant Tween 80

Our data show that fluorescent terbinafine-loaded TFVs rapidly enter the fungal cell. To do this, the vesicles have to overcome the extracellular fungal cell wall and the underlying plasma membrane. The fungal cell wall is a meshwork of cross-linked polysaccharides and manno-proteins [66]. In *Cryptococcus neoformans*, the cell wall restricts passage of macromolecules larger than 270 kDa, which corresponds to a pore size of ∼10 nm [67]. Microscopic methods have shown that most pores are even smaller than this and only few openings extended to 30 nm [68]. The TFVs have an average diameter of ∼90 nm (Dr. U. Vierl, C.P.M. ContractPharma GmbH & Co. KG, Feldkirchen-Westerham, Germany, pers. communication) and therefore the fungal cell wall is an almost insurmountable obstacle for the vesicular drug carriers. However, the results presented in this study show that small fluorescent terbinafine-loaded TFVs rapidly overcome this barrier and enter the fungal cell. This is most likely related to their ability to deform, which is due to the presence of membrane-softening surfactants [1], [5], [6]. Indeed, we show that reduction of the non-ionic surfactant Tween 80 abolished uptake of TFVs. Instead, the drug carriers attached to the cell wall and formed patch-like fields of vesicles that appeared to make contact with the surface but failed to pass through the cell wall. This result suggests that the Tween 80 concentration is important in penetrating hyphae. Most likely, the high concentration of Tween 80 is required to allow ultra-deformation of the vesicles.

One other scenario need to be considered. Atomic force microscopy studies on cell walls of living budding yeast cells have demonstrated the presence of very large pores ranging from 50–250 nm [69]. In addition, evidence for outward transport of intact vesicles through the cell wall of *C. neoformans* exist [26], suggesting that the fungal cell wall is more permeable than currently anticipated [overview in 27], which would allow the passage of TFVs without extreme deformation. We were not able to observe vesicles or pores in the cell wall of *U. maydis*, which makes such extracellular membrane trafficking in this fungus unlikely. However, even in the case those TDT 067 vesicles invade via large pores, our results show that presence of Tween 80 is required for cell invasion. In this scenario the detergent might avoid an unspecific interaction of the hydrophilic vesicles with the surface of the fungus, which could prevent passage of the vesicles through the cell wall. Such activity of Tween 80 was reported for *in vitro* assays, where the surfactant minimizes non-ionic adsorption of viruses to membranes, thereby enhancing passage of the particles through nitrocellulose membranes [70].

We were not able to recognize intact TFVs in the cytoplasm of *U. maydis*, which might be due to a rapid digestion of the incoming vesicle (see below). However, the results summarized here demonstrate that Transfersome®-derived lipids accumulate in the cytoplasm of *U. maydis*. This finding strongly suggests that the vesicles, once having passed through the fungal cell wall, overcome the plasma membrane. A standard way of particle uptake into eukaryotic cells is endocytosis, which has also been reported for filamentous fungi [28], [29], [32], [33], [71], overview in [30], [31] and [72]. Endocytosis occurs predominantly at the hyphal tips where F-actin patches are formed that support the formation and uptake of an endocytic vesicle [29], [34]; [ overview in 38]]. This paper demonstrates that entry of TFVs occurs along the whole length of the fungal cell and is F-actin-independent. Moreover, the formation of Transfersome®-derived lipid droplet occurs under ATP-depleted conditions. Thus, Transfersome® entry to the fungus is ATP- and F-actin-independent, which makes the involvement of the endocytic machinery in their uptake unlikely. The question remains how TFVs cross the plasma membrane. At present we can only speculate on this. Non-enveloped viruses overcome host membranes by a mechanism called membrane penetration [73], which allows passage of intact viral particles of ∼50 nm in diameter through membranes in an endocytosis-independent manner [74]. The molecular basis of this mechanism is not understood [75], but we consider it possible that TFVs could adapt similar strategies to overcome the fungal plasma membrane.

### Cytoplasmic lipases release terbinafine from the Transfersome® vesicles

**Figure 8:**
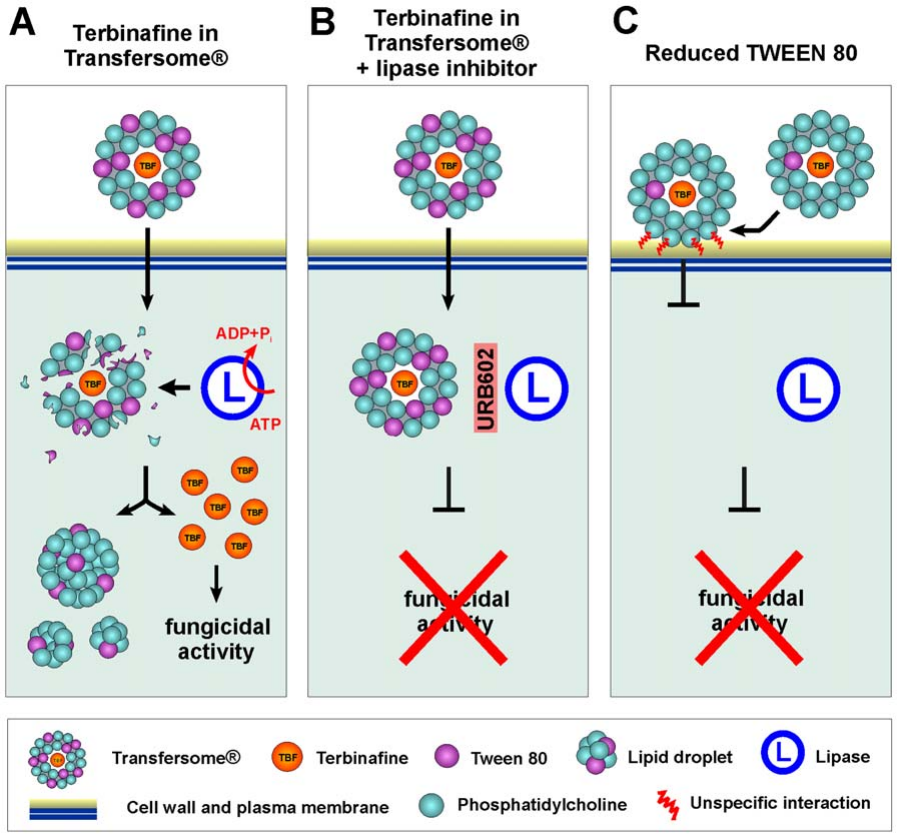
Working model of the mode of action of terbinafine-loaded TFVs in fungal cells. (**A**) Terbinafine-loaded TFVs contain Tween 80 (purple structures) at high concentration. Tween 80 reduces unspecific interaction with the cell wall and allows extreme deformation of the vesicle membrane, thereby allowing entry of intact TFVs. Once inside the cytoplasm, ATP-dependent cytosolic lipases attack the TFVs and might metabolize Tween 80. This results in collapse of the Transfersome® membranes and the formation of lipid droplets. The digestion of the Transfersome® membranes releases terbinafine in the fungal cell. (**B**) In the presence of a lipase inhibitor, lipases cannot release the antifungal. Consequently, terbinafine-loaded TFVs are no longer cytotoxic. (**C**) At lower levels of Tween 80 (phosphatidylcholine to Tween 80 ratio of 20∶1 shown here), terbinafine-loaded TFVs enter at much lower rates. Consequently, the vesicles are not degraded and are not cytotoxic.

Once in the cytoplasm, the terbinafine-loaded TFVs have to release the terbinafine to exert its antifungal activity. In ultra-structural studies, vesicular structures in the fungal cytoplasm were ever observed, but instead globular bodies were seen that are most likely lipid droplets. Similar bodies were reported for dermatophytes treated with TDT 067 [20]. In our experiments, these lipid droplets concentrate the Transfersome® membrane-associated fluorescence and appear shortly after application of the vesicles. However, the increase in fluorescence follows Michaelis–Menten kinetics, suggesting that the presence of the lipid bodies is related to enzymatic activity. Indeed, when ATP levels were reduced by treatment with the ionophore CCCP [45], significantly more lipid-associated fluorescence was detected, which confirms the notion that active cellular processes limit the amount of TFVs in the cell. In pulse-chase experiments, where the supply of TFVs was removed after an initial phase of treatment, the amount of lipid droplets decreased with time. This strongly argues that Transfersome®-derived lipid droplets are digested by fungal lipid-degrading enzymes. An important class of lipid-hydrolyzing enzymes are lipases, which are prominent in fungi [47], [48]. The genome of *U. maydis* contains 21 predicted lipases, among which we found numerous candidates for cytosolic lipases, including homologues of the cytosolic hormone-sensitive lipase (um06274) or phospholipase A2 (um11026). Indeed, the monoacylglycerol inhibitor URB602 [51], [52] prevented the degradation of the lipid bodies and neutralized the antifungal effect of terbinafine-loaded TFVs. The most likely explanation for these results is that fungal lipases attack the incoming TFVs by metabolizing their lipids. This activity releases the enclosed terbinafine and also leads to the aggregation of the remaining membrane fragments into lipid bodies (**Fig. 8**). This process might also be dependent on the concentration of Tween 80, as the surfactant is a substrate for lipases and esterases [76].

### Conclusions

Terbinafine in TFVs (TDT 067) has largely enhanced antifungal activity on dermatophytes compared with conventional terbinafine [18], [20]. The data presented here on terbinafine-induced disruption of the endoplasmic reticulum integrity confirm these results, and they show that this applies to fungi in general. We also report that the surfactant Tween 80 is key for uptake of TFVs into the fungal cell. This might be due to its ability to soften the membrane of the vesicles, which allows ultra-deformability and passage through the small pores of the fungal cell wall. In addition, Tween 80 might help to reduce unspecific binding of TFVs to the fungal cell wall. Once inside the cell, the TFVs are attacked by cytosolic fungal lipases that may use the Tween 80 and phosphatidylcholine components of the Transfersome® as substrates (**Fig. 8**). This metabolic activity releases the enclosed antifungal from the TFVs. Thus, the specific properties of these vesicle carriers do (a) deliver water-insoluble cargo (such as the fungicide terbinafine), (b) allow passage through the fungal cell wall and (c) release their drug content due to the activity of cytosolic fungal lipases. In other words, TFVs act as “Trojan Horses” that efficiently deliver their cytotoxic cargo into the fungal cell. It is important to note that this mode of action does neither depend on the fungal pathogen, nor on the enclosed drug cargo. Therefore, the Transfersome® technology promises novel and powerful ways to address infectious diseases caused by fungi.

## Methods

### Strains and plasmids

The effects of terbinafine and terbinafine-loaded TFVs on the organization of fungal cells were investigated in strains of *U. maydis* expressing organelle-specific GFP markers. The genotypes of all *U. maydis* strains used in this study are listed in **Table 1**.

Strain AB33GRab5a expresses a fusion protein of GFP and the endosome-specific small GTPase Rab5a [32]. In strain AB33ERG, the endoplasmic reticulum is labeled by GFP fused to a targeting sequence and a C-terminal retention signal [24]. Strain AB33GSKL expresses a fusion protein of GFP and peroxisome-targeting signal (SKL [42]). Strain AB33LgaG expresses the mitochondrial protein Lga1 [77]. In strain AB33CPYG, vacuoles are labeled by fluorescent carboxy-peptidase Y [42]. In strain AB33nG, the nuclei contain a fusion protein of GFP and a nuclear localization signal [78].

The effect of the ionophore CCCP on endogenous ATP levels was investigated using strain AB33G_3_Dyn2, in which the dynein heavy chain is labeled by a triple-GFP tag [79], allowing the ATP-dependent observation of the motility of single dynein motors [46]. The plasmid poCpYR [42] was digested with *Nco*I and *Hpa*I to remove the triple mRFP tag which was replaced by a single GFP, cut out of the cloning vector P123 using *Nco*I and *Hpa*I resulting in plasmid poCpYG. For transformation, the plasmid was digested with *Mcs*I and *Hpa*I and transformed ectopically in strain AB33.

### Growth conditions

*U. maydis* liquid cultures were grown overnight in complete medium containing 1% (w/v) glucose, shaking at 200 revolutions per minute at 28°C. The yeast-like cells were harvested by centrifugation at 3000 rpm in a desktop centrifuge and shifted to filamentous growth by transferring them into nitrate-containing minimal medium, as described previously [80]. All assays were conducted at 10–14 h after induction of filamentous growth. Cell wall-less protoplasts were prepared by removing the cell wall of Transfersome®-treated cells, as previously described [81].

### Microscopy

Cells were observed using an IX81 motorized inverted microscope (Olympus, Hamburg, Germany), equipped with a VS-LMS4 Laser-Merge-System with solid state lasers (488 nm/70 mW, Visitron System, Munich, Germany) and a PlanApo 100×/1.45 Oil TIRF objective (Olympus, Hamburg, Germany). Transfersome®-associated fluorescence was observed using the HBO illumination of the IX81 microscope and standard DAPI filter sets (377±50/406/447±60). Images were acquired using a Photometrics CoolSNAP HQ2 camera (Roper Scientific, Germany). All parts of the system were under the control of the software package MetaMorph (MDS Analytical Technologies, Winnersh, UK). For lipid staining, cells that were treated with standard fluorescent TFVs were incubated for 10 min in 1 µM Nile Red (Sigma-Aldrich Ltd, Gillingham, UK; stock 10 mM in methanol).

For transmission electron microscopy, hyphal growth of strain AB33 was induced overnight, as described previously [80], and the cells were incubated with fluorescent terbinafine-loaded TFVs (for details see below). Cells were sedimented by centrifugation at 3000 rpm on a desktop centrifuge and pellets were fixed in glutaraldehyde phosphate buffer (2.5% (v/v) glutaraldehyde in 0.1 M phosphate buffer, pH 7.4) overnight. After three washes in 1 M phosphate buffer (pH 7.4), cells were incubated for 1 h in 1% osmium (w/v) in phosphate buffer, followed by three washes in distilled water. The fixed pellets were incubated for 45 min in 2% uranyl acetate (w/v in water) and after two washes in distilled water cells were dehydrated by 15-min washes in 70%, 80%, 90%, and 100% ethanol. After two additional washes in 100% ethanol, pellets were incubated overnight in a 50∶50 solution of 100% ethanol: Spurr resin (w/v; Agar Scientific, Stansted, UK). Subsequently, the pellets were transferred to 100% Spurr resin overnight, and embedded by incubation at 60°C for 2 days. Ultra-thin sections were cut and placed on nickel grids (Agar Scientific, Stansted, Essex, UK), followed by staining with lead citrate [82]. Samples were investigated using a Jeol JEM 1400 transmission electron microscope (JEOL, Ltd., Welwyn Garden City, UK).

For scanning electron microscopy, either Transfersome®-treated hyphal cells (15–25-min incubation) or 20–50 µl of concentrated Transfersome® suspension was placed on 0.22 micron polycarbonate membrane and air dried for 2 min. Subsequently, the samples were attached to a cryo-sledge and rapidly frozen in liquid nitrogen, followed by water sublimation at −95°C for 3 min using the Alto 2100 chamber (Gatan Ltd., Oxfordshire, UK). This was followed by gold sputtering and observation in Jeol JSM-6390LV scanning electron microscope (JEOL, Ltd., Welwyn Garden City, UK).

### Transfersome® uptake assays

Transfersome® suspension with phosphatidylcholine to Tween 80 ratio (2∶1; identical with the previously reported TDT 067 [20], but labeled with fluorescent 1,6-diphenyl-1,3,5-hexatriene) and Transfersome® suspensions with various phosphatidylcholine to Tween 80 ratios (5∶1, 10∶1, 20∶1) were obtained from IDEA AG (Munich, Germany). All TFVs were loaded with 15 mg/ml terbinafine (AminoChemicals Ltd., Malta) and their membranes were fluorescently labeled with 1,6-diphenyl-1,3,5-hexatriene [25] (DPH; Molecular Probes, Invitrogen Corporation, Grand Island, NY, USA) or DiO (Molecular Probes, Invitrogen Corporation, Grand Island, NY, USA; DiO was used to acquire data for **Movie S2**). For all uptake assays, 10% (v/v) of fluorescent terbinafine-loaded Transfersome® suspension was added to logarithmically growing cells of *U. maydis* (see **Table 1** for detailed strain description). A volume of 1 ml of Transfersome®-exposed cell suspension was incubated on a turning wheel at room temperature for 20–30 min (unless otherwise noted). Cells taken from these assays were placed on 2% agar cushions and images were taken using a HBO illumination devise of the IX81 Olympus microscope at 1 s exposure time. To compare the effect of pure terbinafine and terbinafine-loaded TFVs, was added at an effective concentration of 20–100 µg/ml. In a parallel experiment, the respective concentration of terbinafine enclosed in TFVs was applied which resulted in a much reduced amount of Transfersome® suspension in these experiments (around 0.3% v/v). For request of Transfersome® suspensions used in this study for research purposes contact Sam Yurdakul (Celtic Pharma Development Services Europe Ltd., London, UK ; Email: sam.yurdakul@dev.celticpharma.com).

### Inhibitor treatments

To investigate the uptake of TFVs in the absence of F-actin, fungal cells were pre-incubated in latrunculin A at 10 µM (stock: 20 mM in DMSO: Biomol, Exeter, UK) for 15–30 min. Subsequently, 10% of standard fluorescent terbinafine-loaded Transfersome® solution was added and cells were incubated for an additional 20–30 min. The cells were then placed onto a 2% agar cushion containing the latrunculin A and directly observed under the microscope. Images of blue fluorescence and green fluorescence were taken as described above. The respective volume of DMSO was used in all control experiments. For experiments shown in Figure 3C, LatA and DMSO treated cells were briefly incubated with the endocytic marker dye FM4-64 (Molecular Probes, Leiden, Netherlands), as previously described [33].

For ATP-depletion experiments, cells were pre-incubated for 15 min with 100 µM CCCP (carbonyl cyanide m-chlorophenyl-hydrazone; Sigma-Aldrich Ltd, Gillingham, UK), treated with 10% standard fluorescent terbinafine-loaded Transfersome® suspension, and observed on agar cushions supplemented with 100 µM CCCP. For pulse-chase experiments, cells were treated with standard fluorescent terbinafine-loaded Transfersome® suspension for 30 min, sedimented by centrifugation at 3000 rpm in a desktop centrifuge, and re-suspended in Transfersome®-free medium. After a second centrifugation and resuspension in fresh medium, a sample was immediately investigated at the microscope and again after an additional 30 min turning at room temperature in a 2 ml reaction tube. The respective volume of DMSO was used in all control experiments.

To test for inhibition of lipases, 900 µl of cells were pre-incubated for 30 min with the lipase inhibitors Orlistat (stock at 20 mM in DMSO; Sigma-Aldrich Ltd, Gillingham, UK) or URB602 (stock at 20 mM in DMSO; Sigma-Aldrich Ltd, Gillingham, UK), turning in a 2 ml reaction tube at room temperature. Subsequently, 100 µl of fluorescent standard terbinafine-loaded Transfersome® suspension was added (10% v/v; the condition for a standard uptake assay). The respective volume of DMSO was used in all control experiments.

### Quantitative analysis of Transfersome®-derived fluorescence and image processing

The amount of Transfersome®-associated fluorescence was measured in 14-bit images that were taken as described above. The average fluorescent intensity was measured in sections of hyphal cells that extended 10–15 µm using the software MetaMorph. Intensity scans were performed using the linescan function of the software MetaMorph. Three-dimensional reconstruction of fluorescent lipid bodies (**Movie S2**) was performed using MetaMorph. Images were grey-scaled and processed using MetaMorph. All statistical analyses were conducted using two-tailed T-tests with the software Prism 4 (GraphPad, La Jolla, CA, USA).

## Supporting Information

Figure S1 Effect of terbinafine on the sub-cellular organization in *U. maydis* hyphal cells. Note the fragmentation of mitochondria and the collapse of endoplasmic reticulum in the presence of terbinafine. All organelles were labeled by GFP-marker proteins that are described in the Method section. Bar represents micrometers.
(TIF)

Figure S2 Cell wall-less protoplasts incubated with fluorescent TFVs at 2∶1 and 20∶1 phosphatidylcholine to Tween 80 ratios. TFV-derived fluorescent signals appear in the interior of the rounded cells (**A**). Significantly less TFVs-derived signal is seen at low Tween 80 to phosphatidylcholine ratios (**B**), suggesting that Tween 80 fosters passage through the plasma membrane. Bar represents micrometers.
(TIF)

Figure S3 Localization of fluorescent terbinafine-loaded TFVs (red in both panels, TLT) and marker proteins for vacuoles (carboxy-peptidase Y-GFP; left panel, CPY, green) and early endosomes (GFP-Rab5a; right panel, green). Bar represents micrometers.
(TIF)

Figure S4 Kymographs showing dynein motility in cells treated with the solvent DMSO (control) and with 100 µg/ml of the ionophore cyanide 3-chlorophenyl-hydrazone (CCCP). Motility stopped due to the depletion of ATP. Diagonal lines indicate motility, vertical lines indicate stationary signals. Bars represent seconds and micrometers.
(TIF)

Movie S1 Organization and dynamics of the endoplasmic reticulum in the presence of 30 µg/ml terbinafine in TFVs. Time is given in seconds:milliseconds. The bar represents micrometers.
(MOV)

Movie S2 Three-dimensional reconstruction of fluorescent bodies after 30-min treatment with DiO-labeled TFVs. The bar represents micrometers.
(MOV)

Movie S3 Motility of the motor dynein in DMSO-treated cells (control) and after 45 min in cells treated with the ionophore cyanide 3-chlorophenyl-hydrazone (CCCP). No directed motility of the motors is seen indicating that ATP levels are depleted. Time is given in seconds:milliseconds. The bar represents micrometers.
(MOV)
