# Supplementary figures and images for: Cytoplasmic Fungal Lipases Release Fungicides from Ultra-Deformable Vesicular Drug Carriers
Source: PLoS One. 2012 May 29;7(5):e38181. doi: 10.1371/journal.pone.0038181 (PMC3362563; doi:10.1371/journal.pone.0038181)

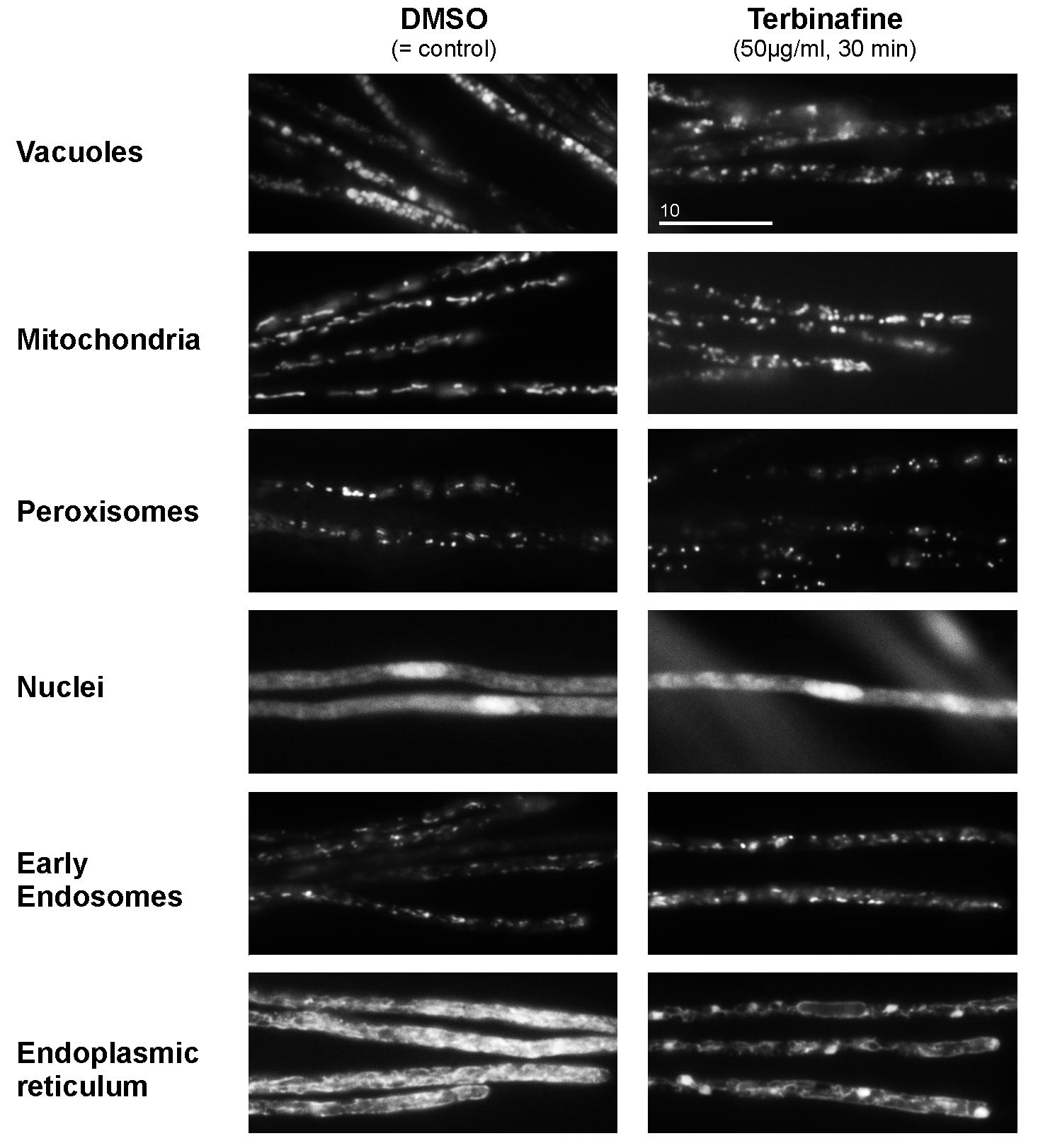

Supplement: Figure S1 — Effect of terbinafine on the sub-cellular organization in U. maydis hyphal cells. Note the fragmentation of mitochondria and the collapse of endoplasmic reticulum in the presence of terbinafine. All organelles were labeled by GFP-marker proteins that are described in the Method section. Bar represents micrometers. (TIF) [file pone.0038181.s001.tif]

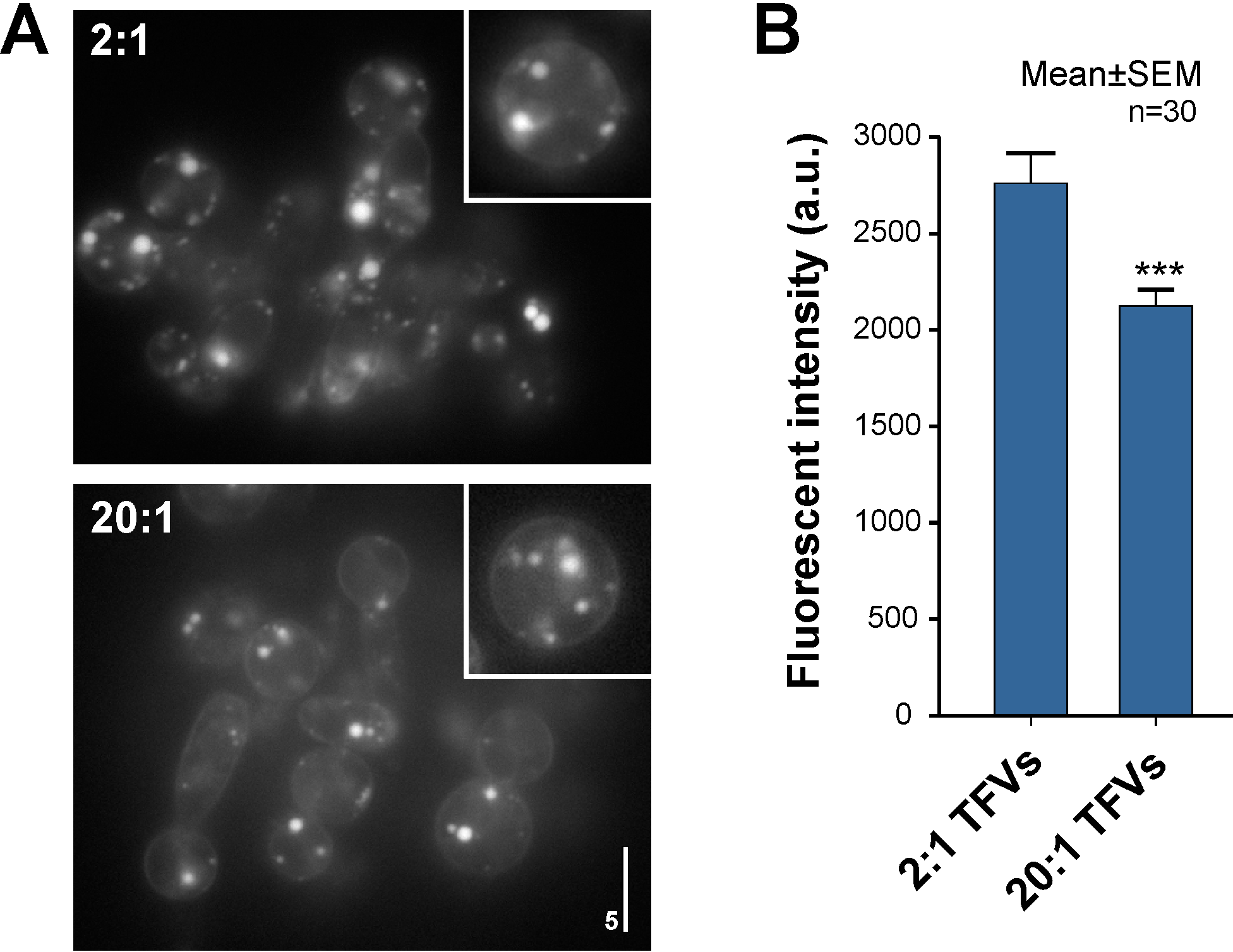

Supplement: Figure S2 — Cell wall-less protoplasts incubated with fluorescent TFVs at 2∶1 and 20∶1 phosphatidylcholine to Tween 80 ratios. TFV-derived fluorescent signals appear in the interior of the rounded cells (A). Significantly less TFVs-derived signal is seen at low Tween 80 to phosphatidylcholine ratios (B), suggesting that Tween 80 fosters passage through the plasma membrane. Bar represents micrometers. (TIF) [file pone.0038181.s002.tif]

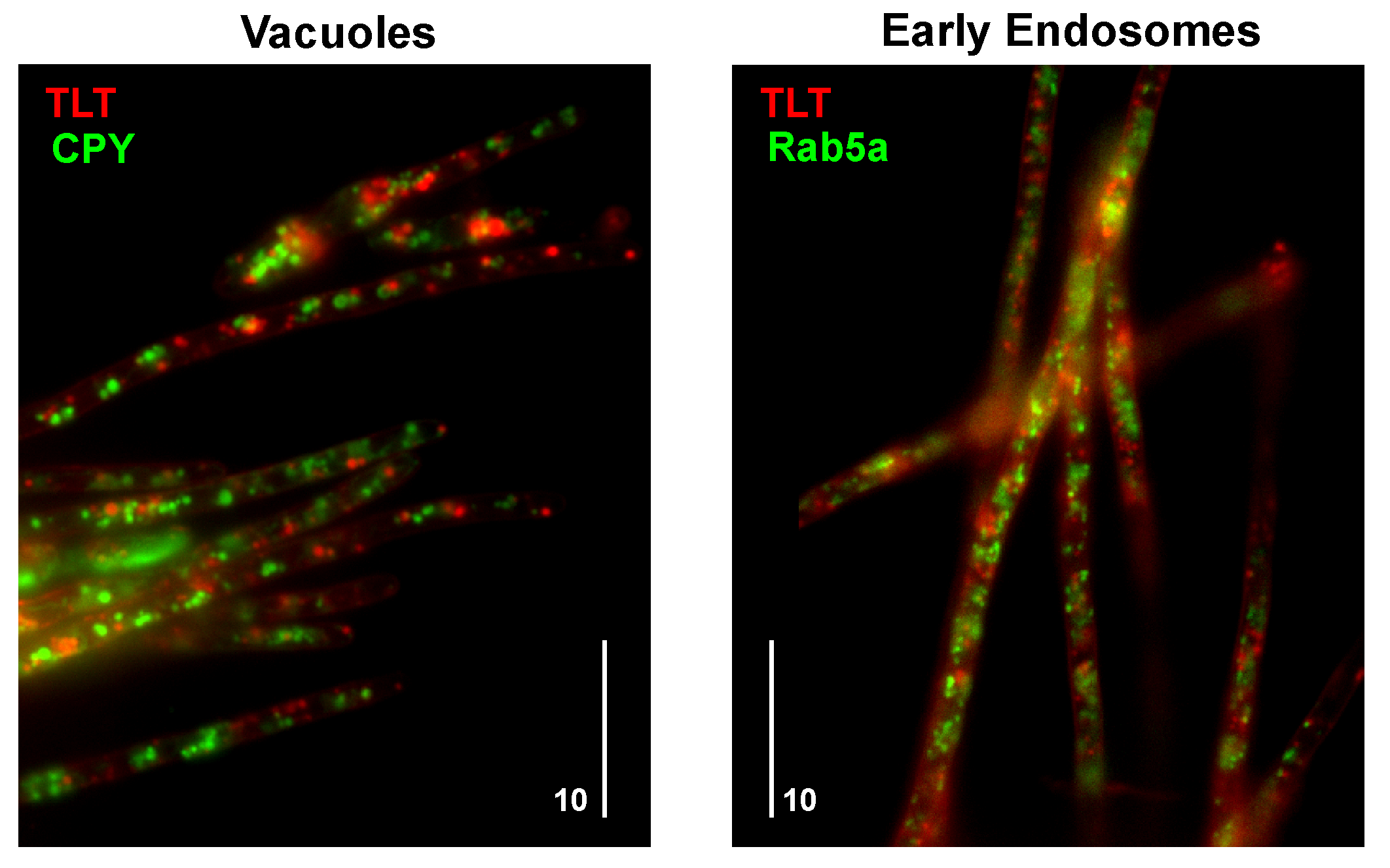

Supplement: Figure S3 — Localization of fluorescent terbinafine-loaded TFVs (red in both panels, TLT) and marker proteins for vacuoles (carboxy-peptidase Y-GFP; left panel, CPY, green) and early endosomes (GFP-Rab5a; right panel, green). Bar represents micrometers. (TIF) [file pone.0038181.s003.tif]

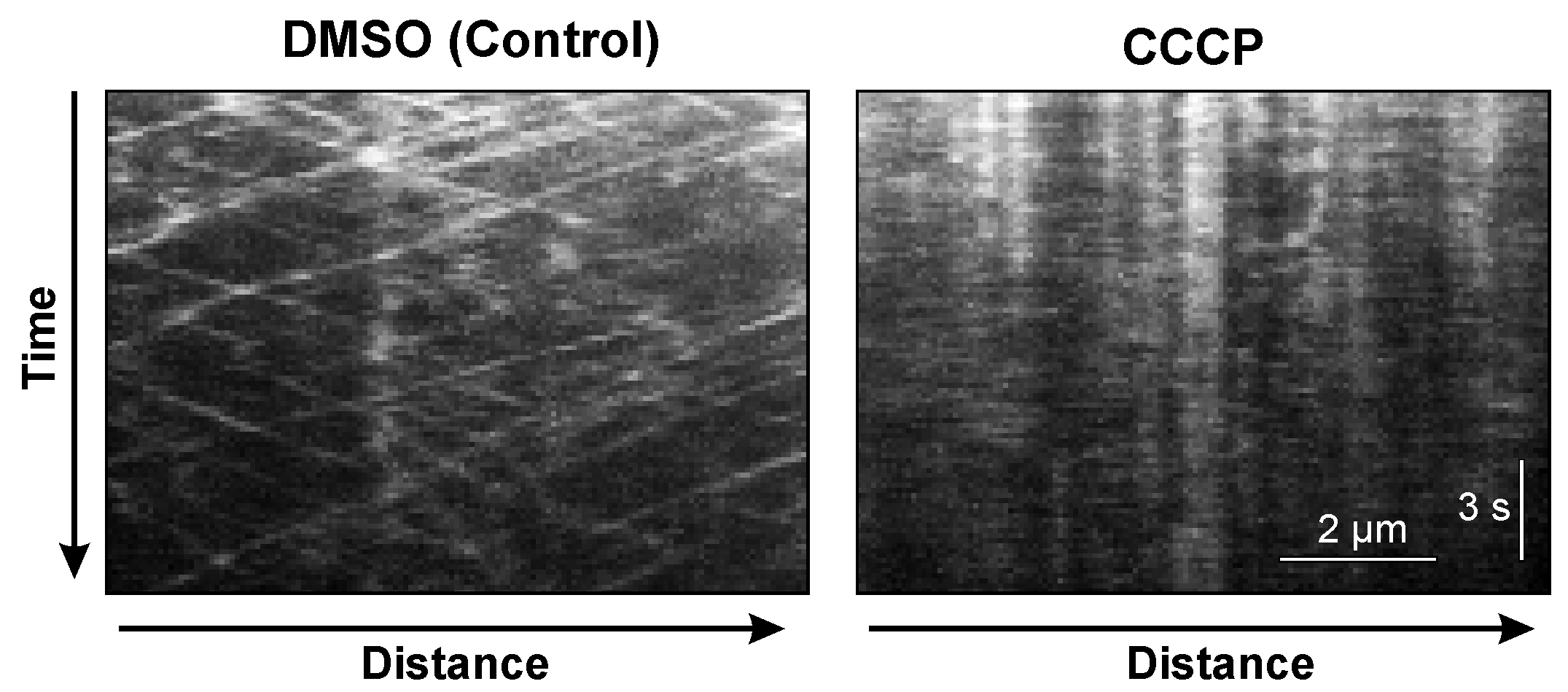

Supplement: Figure S4 — Kymographs showing dynein motility in cells treated with the solvent DMSO (control) and with 100 µg/ml of the ionophore cyanide 3-chlorophenyl-hydrazone (CCCP). Motility stopped due to the depletion of ATP. Diagonal lines indicate motility, vertical lines indicate stationary signals. Bars represent seconds and micrometers. (TIF) [file pone.0038181.s004.tif]
